# Analysis of the effect of cognitive ability on academic achievement: Moderating role of self-monitoring

**DOI:** 10.3389/fpsyg.2022.996504

**Published:** 2022-09-23

**Authors:** Yueqi Shi, Shaowei Qu

**Affiliations:** School of Humanities and Social Sciences, University of Science and Technology Beijing, Beijing, China

**Keywords:** cognitive ability, academic performance, moderating effect, structural equation modeling, self-monitoring

## Abstract

In this study, cognitive ability was classified into memory ability, representational ability, information processing ability, logical reasoning ability, and thinking conversion ability, and analyzed the effects of these five ability values on academic achievement. Structural equation modeling (SEM) was used to analyze the moderating effect of Self-monitoring between cognitive ability and Academic Achievement, using students’ Self-monitoring as moderating variables. The results of the study showed that cognitive ability can significantly and positively affect academic achievement, while Self-monitoring can significantly moderate the effect of cognitive ability on academic performance, with a significant moderating effect on math subjects and English subjects among achievement subjects, and the higher the value of cognitive ability, the stronger the moderating effect.

## Introduction

In China’s educational student evaluation system, colleges and universities usually classify students by their Academic Achievement. Liu (2019) found that Academic Achievement, especially in school-organized examinations, significantly affects the future development of students. Li (2019) found that Academic Achievement is a concentrated expression of student learning and practice, an expression of students’ cognitive abilities, and the particulars of their learning profile and standard. In China’s student growth evaluation system, academic achievement is almost the only reference for admission to colleges and universities, and is the most important goal of student learning. Many existing studies have identified the necessary role of cognitive abilities when it comes to student learning. Therefore, in teaching practice, teachers pay particular attention to the development and exploitation of students’ cognitive abilities. However, other research has found that cognitive ability is not the single major determinant of students’ academic achievement (Shao, 1983). Students’ Academic Achievement may not depend exclusively on the level of cognitive ability, but is determined by a combination of their overall learning status.

However, little research has been done on this part of the study and on the mechanisms of the influence of cognitive ability on Academic Achievement.

This research analyzed the role of cognitive ability on students’ academic achievement under the moderating effect of self-monitoring, using students’ self-monitoring as moderating variables.

### The single effect of cognitive ability

Cognitive ability refers to the human brain’s ability to store memory, process and extraction of information, includes attention, memory and logical reasoning, and thinking transformation. This is a key mental quality of students’ completion of learning activities (Sternberg and Sternberg, 2009), it is also among the most investigated and most reliable predictive factors of student academic achievement (Matthias et al., 2016; Vilia et al., 2017). Xu and Li (2015) found that concentration, working memory, and logical reasoning were significantly predictive factors of Chinese and math achievement in a research of 4,743 middle school students. Rohde and Thompson (2007) concluded that cognitive ability was a direct predictor of academic achievement, with a correlation of 0.38. Ian et al. (2006) found a correlation between cognitive ability and Academic Achievement of 0.81. In a study that followed over 70,000 UK students for 5 years. Paul performed a stepwise analysis by multivariate analysis to obtain normalized regression coefficients (β) to analyze the association between the dimension of logical reasoning and students’ performance in science and chemistry over the three semesters, and found a comparable absolute relationship between reasoning ability and students’ performance in science and chemistry (Grass et al., 2017). Liu measured the cognitive abilities of spatial imagery, computation, and information processing in 499 Chinese children and teamed up to correlate students’ Academic Achievement in mathematics and Chinese over two consecutive school years and found considerable associations between visual-spatial imagery, computation, and information processing abilities and Academic Achievement (Liu et al., 2021). Most of the previous studies of this type have been on the single effect of cognitive ability on Academic Achievement at the individual student level (Kuncel et al., 2004; Miriam et al., 2011). Chen (2016) argues that cognitive ability has a significant impact on the future direction of students, students with strong cognitive ability more likely to attend general high school and those with weak cognitive ability only going to vocational school. In addition, the above findings support the knowledge process theory (Deary et al., 2006; Xu and Li, 2015), that is, the stronger the cognitive ability of students, They are able to extract key information more quickly and accurately and encode it accurately and efficiently in their memory, allowing the brain to output more and more effective information, resulting in greater academic achievement on exams (Liu and Wang, 2000; Zhang and Zhang, 2011). Conversely, at lower levels of cognitive ability, some knowledge would be missed during the knowledge process, which would further reduce the effective information output and lead to lower academic achievement (Miriam et al., 2011). These findings also support the results of the previous analyses, suggesting that cognitive abilities typically contribute significantly to academic achievement.

While the correlation is clear between students’ cognitive ability and academic achievement, it remains very challenging to clarify the complex mechanisms that influence them.

Through the research of many scholars, we can find that the association between students’ cognitive abilities and academic achievement is clear, but the mechanisms of their complex influence remain very ambiguous. The cognitive abilities are important in students’ study activities only through the researcher’s predetermined scope of investigation that includes particular cognitive abilities, but outside the survey, these cognitive abilities are still operating in unpredictable ways (David, 2005), thus, the scholars still have not reached a consensus on the reasons why cognitive ability affects academic achievement due to the inconsistency in investigating the range of students’ cognitive abilities (Formazin et al., 2011). The study by Xu and Li (2015) concluded that there was a significant correlation between attention and academic achievement, with the correlation coefficient ranging between 0.4 and 0.5. However, Zhang (2008) found that logical reasoning ability (LRA) had a correlation coefficient of around 0.3 with both Chinese and math scores, while attention was not significantly related to either subject. These findings all support the fact that cognitive ability can influence academic achievement indirectly, but this still does not lead to accurate conclusions about the advanced effect of individual student factor on academic achievement.

### The moderating role of self-monitoring

Many previous studies have shown that there is indeed a link between the academic achievement of students with the personality factors of them. Gerhard (1996) concluded from an empirical analysis and concluded that personality traits contribute significantly to the academic achievement of students. Leino (1989) considered that non-intelligent factors and personality traits are the major causes of academic achievement. Gough (1964) studied 18 personality factors in secondary school students using the California Psychological Inventory (CPI) and correlated them with Academic Achievement and found that at least eight personality factors (e.g., control, responsibility, tolerance, and independence) were significantly related to Academic Achievement. Richardson et al. (2009) found that both rigor and achievement motivation among personality traits were significantly correlated with students’ GPA by measuring five major personality traits and achievement motivation, and that this effect was moderated by achievement motivation. Adrian and Tomas (2005) analyzed the correlation between students’ personality traits and their level of knowledge and showed that the rigorous and open personality traits were significantly correlated with the level of knowledge.

Self-monitoring, as a behavioral manifestation external to personality traits, also directly affects academic achievement. Callan and Cleary (2019) found that self-monitoring showed a significant positive correlation with math achievement and was a unique predictor of student achievement through an analysis of 96 8th grade students. Sabry (2010), through a follow-up study of 119 students found that self-management and self-monitoring significantly predicted students’ academic achievement. Sawhney and Bansal (2015) found significant differences in academic achievement between students with strong and weak self-monitoring through a study of 100 students.

On the correlation between cognitive abilities and self-monitoring, many scholars have done research, but it is mainly biased toward qualitative research or analysis of the correlation between the two, so the interaction relationship between them has been little studied. Cognitive ability and self-monitoring are measured using a variety of dimensions, so the analysis of their correlations has different conclusions (James et al., 2006). Cognitive ability and self-monitoring are both separate and interrelated elements of individual student psychology (Li and Zhang, 2015).

Most self-monitoring are minimally correlated with cognitive ability, and therefore, cognitive ability and self-monitoring are often used as independent variables that affect students’ academic achievement (Roemer et al., 2022); However, some scholars have argued that cognitive ability and self-monitoring interact (Borghans et al., 2008), Wang (2004) found through a study that self-monitoring in metacognition enhances students’ learning ability and compensates for learning difficulties due to uneven cognitive development. Hayat and Shateri (2019) found through a study of 225 students that learning ability has a direct and positive effect on self-monitoring in metacognitive strategies. Zhang and Wei (2012) found that good self-monitoring strategies help to enhance students’ learning ability by examining the relationship between students’ self-monitoring strategies and their self-directed learning ability.

The inconsistent findings of the above studies may be related to the fact that Self-monitoring play more of a moderating role in Academic Achievement. Among the effects of cognitive ability on academic performance, the positive moderating effect of self-monitoring was found to be significant (Zhu et al., 2017), and that higher indices of self-regulation in Self-monitoring enable students to focus more on tasks and achieve better Academic Achievement (Nesayan et al., 2019), and self-regulation also significantly enhances the positive effect of cognitive ability on academic achievement (Shi and Qu, 2021).

### Research hypothesis

In this study, cognitive abilities were classified into memory ability (MA), information processing ability (IPA), logical reasoning ability (LRA), representational ability (RA), and thinking conversion ability (TCA) based on the classification of cognitive abilities by Wo and Lin (2000) and Liang et al. (2020). Cognitive abilities will be analyzed for their role in influencing academic achievement and the following hypotheses will be formulated.

**Hypothesis 1:** Cognitive abilities can influence academic achievement positively.

Meanwhile, the available research still analyses the effects of cognitive ability and self-monitoring as separate factors on students’ academic achievement, while the effect of both together on academic achievement is unclear. Regarding the relationship between cognitive ability and self-monitoring on academic achievement, several empirical studies have shown that self-monitoring can significantly moderate the relationship between the influence of cognitive ability on students’ knowledge, Freeman et al. (2017) found by examining the relationship between working memory capacity, self-monitoring, and academic achievement in 73 4th grade students that self-monitoring can influence the performance of working memory capacity and is reflected in and in academic achievement. Alberto et al. (2021) found in a study of 133 students aged 6–9 years that working memory capacity was not significantly different in its effect on overall achievement and grades in language and mathematics subjects. Leue et al. (2014) investigated the relationship between self-monitoring and the cognitive abilities of working memory, logical reasoning, language, and representational skills. The results found that self-monitoring and the moderation of working memory capacity were the most prominent, and self-monitoring also had significant moderation effects on logical reasoning capacity and representational capacity. This research hypothesizes that self-monitoring can also significantly and positively moderate the influence of cognitive ability on academic achievement and proposes the following hypotheses.

**Hypothesis 2:** A positive moderating effect of self-monitoring between cognitive ability and academic achievement.

## Materials and methods

### Participants

The ethical examination of this study was approved by the Research Ethics Committee of the School of Humanities and Social Sciences, University of Science and Technology Beijing, and the study was conducted in accordance with the regulations for the protection of human subjects. This study selected 569 students as samples, all aged 15–18 years old, As shown in Table 1.

**TABLE 1:** Distribution of participating students.

Grade	Number of students			
	Boys	Proportion	Girls	Proportion
First grade	114	50.89%	110	49.11%
Second grade	82	46.33%	95	53.67%
Third grade	92	54.76%	76	45.24%
Total	288	50.62%	281	49.38%

### Procedure

Both the cognitive ability and personality trait measures in this research were conducted on campus. The students who took the test were organized by staff and tested in a separate classroom. The entire test lasted for 2 h.

Structural equation models were developed based on the cognitive ability, self-monitoring ability and academic achievement of the students obtained, and comprehensive achievement models and sub-subject models for Chinese, mathematics and English were developed, respectively. Each model was first tested for common method deviation in the analysis process, and then the fit of the model was tested according to the CFA test process. Finally, the influence of cognitive ability on academic achievement and the significance of the moderating effect of self-monitoring were analyzed according to the structural equation model, and the moderating effect was specifically analyzed using a simple slope test.

### Measures

#### Cognitive ability

A stimulus-informed cognitive ability system designed by Wo (2010) was used for the cognitive ability test. The test uses computerized testing methods with techniques such as subtractive response times and additive response times (accurate to nanoseconds). The total number of correct fixed points of student feedback and the fixed feedback time were recorded throughout the testing process, and the accuracy of students’ cognitive ability was analyzed based on the feedback records. The cognitive accuracy of the tested students was obtained by statistical methods, and their corresponding cognitive ability values were quantified and the quantified values were converted into T-scores to obtain the final cognitive ability values of the tested students. The final cognitive ability values contain five ability values: MA, LRA, RA, IPA, and TCA. The test method has been patented as an invention, and the sample size of the general test exceeds 2 million. The values of students’ cognitive abilities obtained from the test were normally distributed with a range of trends of ±50 centered at 100, with high discriminant validity. The Cronbach’s alpha of the test ranged among 0.80–0.90.

#### Self-monitoring

The Student Personality Trait Scale was designed by Zhang et al. (2005). The scale uses a 5-point Likert scale: 5 (very much the same), 4 (comparatively the same), 3 (uncertain), 2 (relatively different), and1 (very different). There were forty-eight questions, consisting of four dimensions: planning, self-control, persistence and daring, with 12 questions set for each dimension. After the student assessment, the student’s corresponding Self-monitoring dimension value was obtained by accumulating the scores of each dimension, and converted to T-score as a value of the students’ personality trait dimensional ability. The Cronbach alpha coefficient for all the dimensions ranged from 0.60 to 0.93, with a validity of 0.91 and a test-regression reliability of 0.85.

#### Academic achievement

In the current research, in order to reduce the influence due to the level of students’ test performance, the average of the students’ four test scores in the semester when the cognitive ability was tested to be worthy was used as the academic score for each subject, and the raw scores were standardized (scores were assigned according to levels, with the highest score being 100 and the lowest being 0). In this study, three subjects, Chinese, Math and English, were selected for the study, and the composite academic score was the sum of the three subject’s total scores.

### Data analysis

This research first analyzed the correlation between cognitive ability, self-monitoring, and academic achievement, and then used structural equation modeling to analyze the moderating role of self-monitoring based on the moderating utility modeling procedure suggested by Wen and Ye (2014), and analyzed the pattern of the moderating role through a simple slope test. SPSS 25.0 and Mplus8.3 software were used to analyze the data.

## Results

### Common method deviation test

This study used a questionnaire in which the dimensions and sequence of test questions were set randomly, which reduced the bias introduced by self-designed questionnaires. Harman’s single-factor test was used to test the effects of the procedure (Podsakoff et al., 2003), and an exploratory factor analysis of two variables (cognitive ability, and self-monitoring) was conducted. The results showed that the characteristic roots of all nine factors were higher than 1 after factor rotation, with the variance explained by the first factor being 33.38% (less than 40% of the critical value), so the variation level of the used methods is within an acceptable level (Wang et al., 2016).

### Descriptive and bivariate analyses

The means, standard deviations, and correlation coefficients of each study variable are shown in Table 2. Cognitive ability, self-monitoring, and academic performance all showed significant positive correlations. For more data see Supplementary Table.

**TABLE 2:** Means, standard deviations, and intercorrelations for variables.

	M	SD	1	2	3	4	5	6	7	8	9	10	11	12	13
1. MA	106.58	13.39	1												
2. IPA	105.17	11.396	0.477**	1											
3. RA	107.9	6.812	0.431**	0.464**	1										
4. LRA	106.12	7.937	0.381**	0.429**	0.301**	1									
5. TCA	97.35	14.698	0.483**	0.592**	0.514**	0.483**	1								
6. PLANING	103.37	14.72	0.316**	0.420**	0.163**	0.310**	0.359**	1							
7. SELFCONTROL	107.17	14.27	0.423**	0.485**	0.219**	0.360**	0.423**	0.594**	1						
8. PERSISTENCE	104.66	14.893	0.429**	0.465**	0.192**	0.336**	0.413**	0.674**	0.512**	1					
9. DARING	101.66	15.638	0.430**	0.461**	0.270**	0.346**	0.432**	0.564**	0.609**	0.648**	1				
10. CHINESE	57.81797666	25.72765725	0.449**	0.496**	0.322**	0.390**	0.496**	0.530**	0.545**	0.574**	0.555**	1			
11. MATHEMATICS	50.47993002	28.91223569	0.426**	0.530**	0.319**	0.376**	0.481**	0.465**	0.530**	0.520**	0.502**	0.275**	1		
12. ENGLISH	52.20492511	28.08333079	0.444**	0.396**	0.202**	0.380**	0.397**	0.495**	0.526**	0.528**	0.519**	0.299**	0.117**	1	
13. TS	160.5028318	58.07603715	0.631**	0.681**	0.402**	0.548**	0.657**	0.612**	0.666**	0.675**	0.653**	0.730**	0.683**	0.679**	1

*N* = 569, *p < 0.05, **p < 0.001.

### Measurement model check

An exploratory factor analysis was required to check the quality of the model before conducting the moderating effects analysis. Two latent variables were included for the present research, which were cognitive ability (five indicators including MA, IPA, LRA, RA, and TCA) and self-monitoring (four indicators including planning, self-control, persist, and daring). The test results showed that the model fit was good. χ^2^(26) = 88.084, CFI = 0.973, TLI = 0.962, SRMR = 0.042, RMESA = 0.065, and the 90% confidence interval for RMSEA was [0.050, 0.080], which indicated that the fitted indicators were within the normal reception range. It is also shown in Table 3 that all of the latent variable indicators had significant standardized loadings on the corresponding factors (*p* < 0.001).

**TABLE 3:** Factor loading coefficient table.

Variable	Non-std. (Coef.)	SD	*z* (CR)	Std.
***Cognitive ability***				
MA	1	–	0.833301027	0.650***
IPA	1.004	0.069		0.766***
RA	0.462	0.039		0.591***
LRA	0.532	0.045		0.583***
TCA	1.313	0.091		0.778***
***Personality traits***				
PLANING	1	–	0.886514422	0.743***
SELF-CONTROL	1.06	0.056		0.812***
PERSIT	1.186	0.058		0.871***
DARE	1.089	0.061		0.762***

*p < 0.05, **p < 0.01, and ***p < 0.001.

### Moderating model checking

**FIGURE 1:**
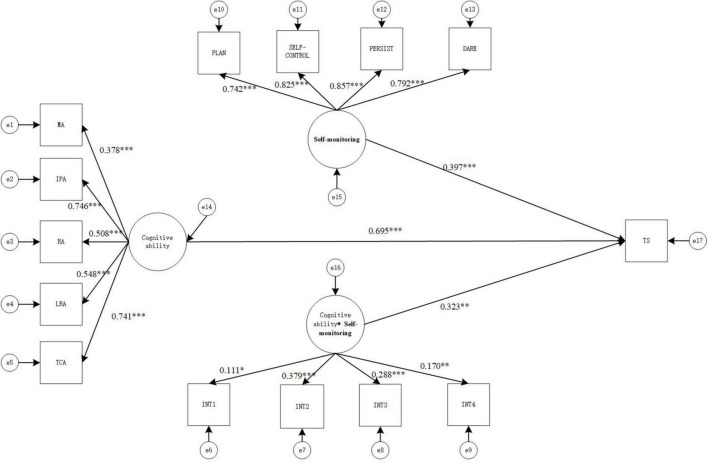
Structural equation moderating effect relationship model diagram (model 1). MA, memory ability; IPA, information processing ability, RA, representation ability; LRA, logical reasoning ability; TCA, thinking conversion ability; TS- academic performance. *p < 0.05, **p < 0.01, and ***p < 0.001.

This study used structural equation modeling for examining the influence of cognitive ability, self-monitoring, and interaction terms on academic achievement, and the results are shown in Figure 1. On the basis of the moderated process of analysis suggested by Wen and Ye (2014), the impact of cognitive ability on academic achievement was analyzed using structural equation modeling. The results showed that cognitive ability significantly and positively influenced academic achievement (β = 0.868, *p* < 0.001). Then, self-monitoring and interaction terms were brought into the model as moderating variables.

#### Model 1: Impact on total academic achievement

The values of the goodness-of-fit indicators of the structural equation model were χ^2^(72) = 332.136, CFI = 0.945, TLI = 0.930, SRMR = 0.073, RMESA = 0.079, the 90% confidence interval of RMSEA is [0.071, 0.088], which shows that the values of the goodness-of-fit indicators of the model were all in the acceptable range and the fit was good. The result is shown in Figure 1.

As seen in Figure 1, cognitive ability has positive and significant correlation with total academic achievement (γ = 0.695, *p* < 0.001), Self-monitoring has positive and significant correlation with total academic achievement (γ = 0.397, *p* < 0.001), and the interaction term also has positive and significant correlation with total academic achievement (γ = 0.323, *p* < 0.005), indicating that the Self-monitoring between cognitive ability and total Academic Achievement moderating effect is significant. Therefore, hypothesis1,2 is valid.

**FIGURE 2:**
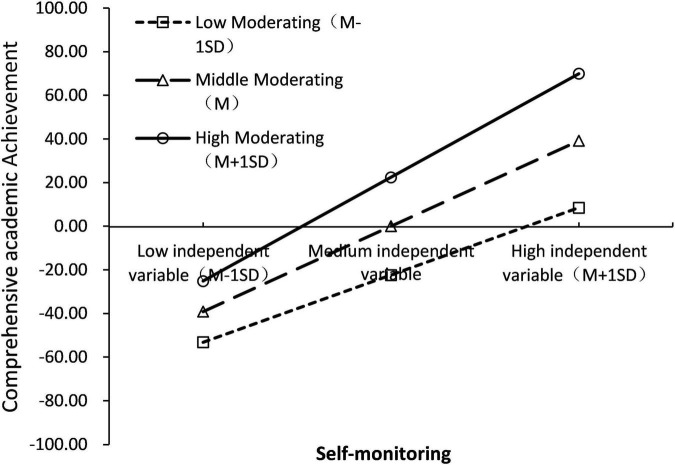
Simple slope test (model 1).

To reveal more clearly the specific pattern of moderating effects, a simple slope test was further used to analyze the moderating role of Self-monitoring. Firstly, subjects were divided into high support group (Z ≥ 1SD), low support group (Z ≤ 1SD), and medium support group (between the above two groups) according to the mean score of Self-monitoring plus or minus one standard deviation; secondly, group regression was used to examine the relationship between cognitive ability and total Academic Achievement, controlling for variables such as gender and grade. The results showed that cognitive ability in all three personality trait groups significantly predicted total Academic Achievement (*p* < 0.001), with a prediction coefficient β of 0.372 for the low Self-monitoring group, 0.695 for the medium Self-monitoring group, and 1.018 for the high personality trait group. The results are shown in Figure 2. As the value of Self-monitoring increases, the prediction of cognitive ability on total Academic Achievement tends to increase gradually; and as the cognitive ability increases, the higher the value of students’ Self-monitoring, the higher the total Academic Achievement.

#### Model 2: Impact on Chinese academic achievement

**FIGURE 3:**
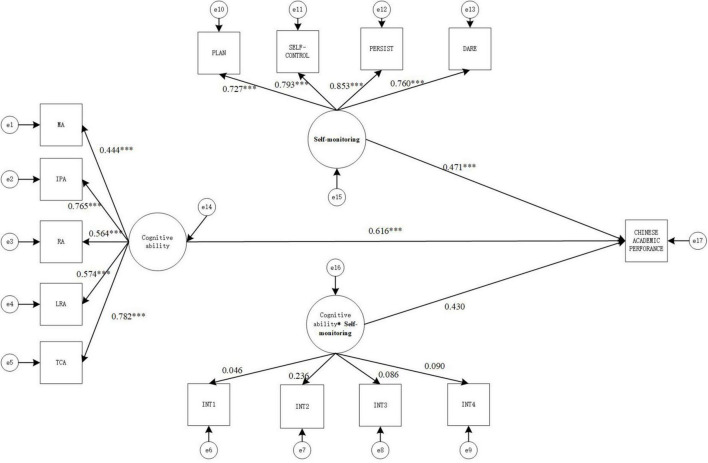
Structural equation moderating effect relationship model diagram (model 2). MA, memory ability; IPA, information processing ability; RA, representation ability; LRA, logical reasoning ability; TCA, thinking conversion ability.*p < 0.05, **p < 0.01, and ***p < 0.001.

The values of the goodness-of-fit indicators of the structural equation model were χ^2^(72) = 468.731, CFI = 0.864, TLI = 0.829, SRMR = 0.100, RMESA = 0.098, the 90% confidence interval of RMSEA is [0.090, 0.107], which shows that the values of the goodness-of-fit indicators of the model were relatively general. The result is shown in Figure 3.

As seen in Figure 3, cognitive ability has positive and significant correlation with Chinese academic achievement (γ = 0.616, *p* < 0.001), Self-monitoring has positive and significant correlation with Chinese academic achievement (γ = 0.471, *p* < 0.001). But the interaction term could not positively predict Chinese Academic Achievement (γ = 0.430, *p* > 0.01), indicating that the Self-monitoring between cognitive ability and Chinese Academic Achievement moderating effect is not significant.

#### Model 3: Impact on mathematics academic achievement

**FIGURE 4:**
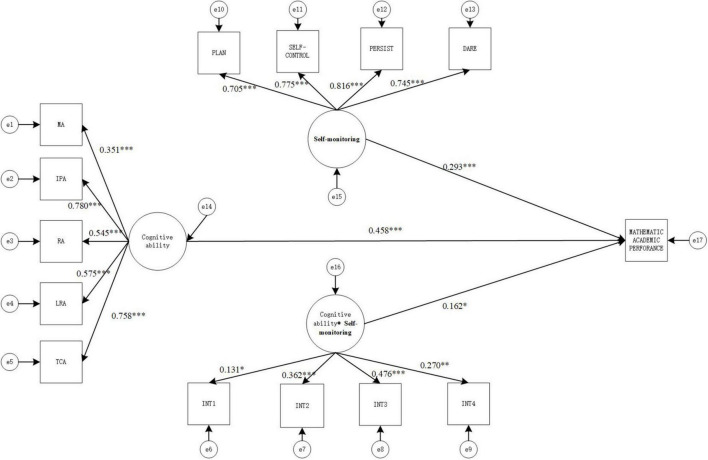
Structural equation moderating effect relationship model diagram (model 3). MA, memory ability; IPA, information processing ability; RA, representation ability; LRA, logical reasoning ability; TCA, thinking conversion ability.*p < 0.05, **p < 0.01, and ***p < 0.001.

The values of the goodness-of-fit indicators of the structural equation model were χ^2^(72) = 337.206, CFI = 0.977, TLI = 0.969, SRMR = 0.039, RMESA = 0.056, the 90% confidence interval of RMSEA is [0.043, 0.070], which shows that the values of the goodness-of-fit indicators of the model were all in the acceptable range and the fit was good. The result is shown in Figure 4.

As seen in Figure 4, cognitive ability has positive and significant correlation with mathematics academic achievement (γ = 0.458, *p* < 0.001), Self-monitoring has positive and significant correlation with mathematics academic achievement (γ = 0.293, *p* < 0.001), and the interaction term also has positive and significant correlation with mathematics academic achievement (γ = 0.162, *p* < 0.005), indicating that the Self-monitoring between cognitive ability and mathematics Academic Achievement moderating effect is significant.

**FIGURE 5:**
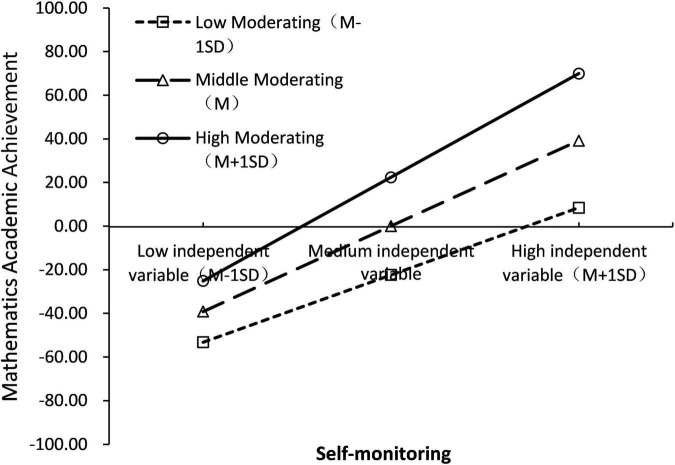
Simple slope test (model 3).

To reveal more clearly the specific pattern of moderating effects, a simple slope test was further used to analyze the moderating role of Self-monitoring. Firstly, subjects were divided into high support group (Z ≥ 1SD), low support group (Z ≤ 1SD), and medium support group (between the above two groups) according to the mean score of Self-monitoring plus or minus one standard deviation; secondly, group regression was used to examine the relationship between cognitive ability and mathematics Academic Achievement, controlling for variables such as gender and grade. The results showed that cognitive ability in all three personality trait groups significantly predicted mathematics Academic Achievement (*p* < 0.001), with a prediction coefficient β of 0.296 for the low Self-monitoring group, 0.458 for the medium Self-monitoring group, and 0.620 for the high personality trait group. The results are shown in Figure 5. As the value of Self-monitoring increases, the prediction of cognitive ability on mathematics Academic Achievement tends to increase gradually; and as the cognitive ability increases, the higher the value of students’ Self-monitoring, the higher the mathematics Academic Achievement.

#### Model 4: Impact on English academic achievement

**FIGURE 6:**
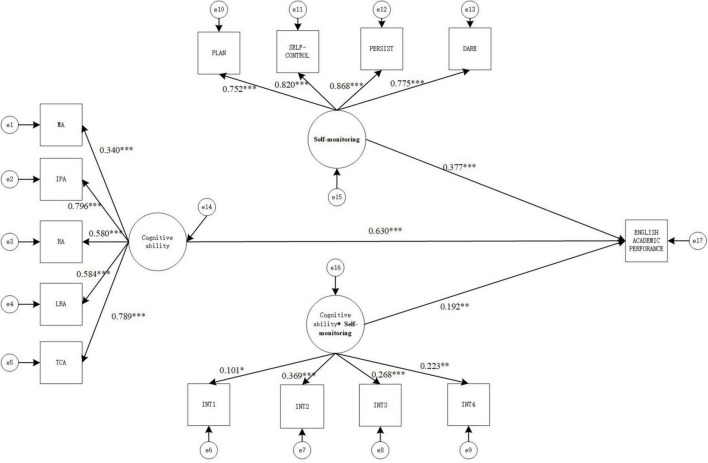
Structural equation moderating effect relationship model diagram (model 4). MA, memory ability; IPA, information processing ability; RA, representation ability; LRA, logical reasoning ability; TCA, thinking conversion ability. *p < 0.05, **p < 0.01, and ***p < 0.001.

The values of the goodness-of-fit indicators of the structural equation model were χ^2^(72) = 384.832, CFI = 0.970, TLI = 0.960, SRMR = 0.042, RMESA = 0.064, the 90% confidence interval of RMSEA is [0.051, 0.077], which shows that the values of the goodness-of-fit indicators of the model were all in the acceptable range and the fit was good. The result is shown in Figure 6.

As seen in Figure 6, cognitive ability has positive and significant correlation with English academic achievement (γ = 0.630, *p* < 0.001), Self-monitoring has positive and significant correlation with English academic achievement (γ = 0.377, *p* < 0.001), and the interaction term also has positive and significant correlation with English academic achievement (γ = 0.192, *p* < 0.005), indicating that the Self-monitoring between cognitive ability and English Academic Achievement moderating effect is significant.

**FIGURE 7:**
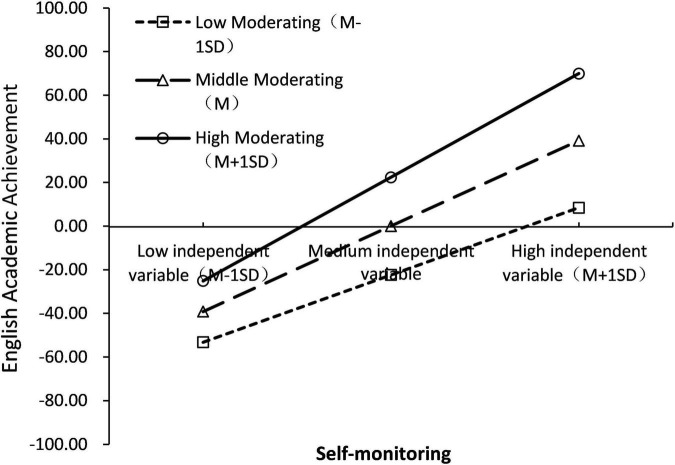
Simple slope test (model 4).

To reveal more clearly the specific pattern of moderating effects, a simple slope test was further used to analyze the moderating role of Self-monitoring. Firstly, subjects were divided into high support group (Z ≥ 1SD), low support group (Z ≤ 1SD), and medium support group (between the above two groups) according to the mean score of Self-monitoring plus or minus one standard deviation; secondly, group regression was used to examine the relationship between cognitive ability and English Academic Achievement, controlling for variables such as gender and grade. The results showed that cognitive ability in all three personality trait groups significantly predicted English Academic Achievement (*p* < 0.001), with a prediction coefficient β of 0.438 for the low Self-monitoring group, 0.630 for the medium Self-monitoring group, and 0.822 for the high personality trait group. The results are shown in Figure 7. As the value of Self-monitoring increases, the prediction of cognitive ability on English Academic Achievement tends to increase gradually; and as the cognitive ability increases, the higher the value of students’ Self-monitoring, the higher the English Academic Achievement.

## Discussion

### The single effect of cognitive ability on academic achievement

Many researches have obtained that cognitive ability is a very important pyschological characteristic of students and that cognitive ability ensures the successful achievement of learning activities (Matthias et al., 2016).

IPA (information processing ability) is mainly the ability of students to understand information after acquiring it through reading and listening, which is closely related to students’ classroom efficiency. Students with better IPA ability can fully understand and master the content taught by the teacher in class and quickly construct it into their own knowledge system, thus enhancing their knowledge mastery and achieving better Academic Achievement in exams (Yuan and Wen, 2003).

TCA (Thinking Conversion Ability) is the active situation of students’ thinking in understanding knowledge during the learning process, which is mainly reflected in the rate and accurately of thinking conversion, therefore, no subject can be learned without the ability. In mathematics, in particular, students with strong TCA ability are more likely to summary and acquire thinking and skills to solve new mathematical questions, and to make analogies in similar problems, thus improving the accuracy of solving novel and difficult problems, and thus improving their academic performance (Liu, 1988).

MA (Memory Ability) is mainly a student’s long term memory ability. The stronger the MA ability value, the more students are able to remember knowledge quickly and keep it for a long time. In addition, MA can interact with IPA to significantly improve students’ reading ability by mastering more reading information through memory ability and thus comprehending information more quickly during reading, which is particularly evident in reading comprehension questions on both Chinese and English exams. As a result, Academic Achievement is also better among students with strong MA proficiency values (Yu et al., 2014).

LRA (Logical Reasoning Ability) includes both types of inductive and deductive reasoning. LRA has a significant positive impact on mathematics academic achievement of students (Zhu et al., 2020). Recently, Chinese higher education entrance examinations have begun to focus on students’ reasoning ability, which is also reflected in the Chinese and English subject examinations, where a large number of logical reasoning questions have been added (Hu, 2017); therefore, a significant positive effect of LRA has also been demonstrated on academic performance in both Chinese and English examinations.

RA (representational ability) helps students to understand three-dimensional spatial knowledge in mathematics subjects by constructing three-dimensional images and pictures in their own minds, which helps to understand spatial knowledge. In addition, RA can also stimulate associative memory during students’ memorization process by forming images in their minds while they recite knowledge related to Chinese and English, which leads to stronger and longer-lasting memory for knowledge and thus better Academic Achievement (Lin et al., 2003).

Meanwhile, we can find that the effect of MA on Academic Achievement in Chinese and English (correlation coefficients of 0.439 and 0.435, respectively) is greater than that on Academic Achievement in mathematics (correlation coefficient of 0.424), while the effect of IPA on Academic Achievement in mathematics (correlation coefficient of 0.531) is much greater than that on Academic Achievement in Chinese (correlation coefficient of 0.496) and English (correlation coefficient of 0.393). It can be surmised that for the subjects of Chinese and English, the test focuses on students’ memorized knowledge, while for the subject of mathematics, the test focuses on students’ knowledge processing and information extraction skills.

### The moderating role of self-monitoring

Significant positive moderating effects of self-monitoring between cognitive ability and academic performance were observed, and the higher the value of students’ cognitive ability, the more pronounced the effect of the moderating component.

Students’ behavior in learning activities is guided by their individual stable self-monitoring by nature, may receive indirect influence from the learning environment, but is essentially determined by their own self-monitoring (Nie et al., 2011). During the learning process, the individual student’s typical response to environmental stimuli is real-time, emotional, and conditioned. However, individual students can prevent the triggering of external stimuli through specific manifestations of self-monitoring so that they do not react impulsively and in a timely manner. Self-monitoring can influence and temporarily alter individual behavioral characteristics by organizational strategies including planning, persistence, self-control, and daring, thereby reducing impulsive behavior and producing other positive learning behaviors in response to situations (Mischel and Mischel, 1983; Mischel and Shoda, 1998).

Usually, those students who have higher levels of self-planning, self-restraint, self-persistence and self-regulation within their self-monitoring are able to keep more stable information emotionally during the learning process in the face of learning pressure from the external environment, thus ensuring that their learning efficiency is not affected by the outside world.

In self-determination theory, good academic behavior outcomes (e.g., Academic Achievement) can occur if students meet specific requirements for psychological trait stability (e.g., persistence and self-discipline) (Ryan and Deci, 2000). In this way, there is a virtuous cycle of learning that students can establish, including planning, self-regulated execution, persistence, and dynamic adjustment, so that they can continuously improve their learning process and achieve better Academic Achievement in examinations (Lin, 2020).

Students with high planning and self-control in their Self-monitoring tend to have more time to study and higher learning efficiency, which allows students to fully utilize their cognitive abilities, thus allowing them to achieve academic satisfaction with less time spent. In addition, the persistence and dare nature of personality characteristic are very helpful for students to challenge learning difficulties, and together with strong cognitive ability, students can easily solve learning problems, thus gaining a greater sense of academic achievement and higher Academic Achievement.

Usually, students with high learning efficiency will also have stronger learning self-confidence; therefore, when students have strong values of Self-monitoring, their learning efficiency will also be more prominent, which is more obvious when students have stronger cognitive ability, the stronger students’ cognitive ability, the higher students’ learning self-confidence, the more willing students are to take the initiative to learn, and thus their learning efficiency will be improved, which in turn will improve their Academic Achievement (Liang et al., 2020). Therefore, as a result, it is clear that Self-monitoring can significantly moderate the effect of cognitive ability on academic performance, and the stronger the cognitive ability, the more prominent the moderating effect.

In addition, in the analysis of the moderating effect of Chinese, mathematics, and English subjects, it can be found that the moderating effect of Self-monitoring is significant for mathematics and English subjects, but not for Chinese subjects. This is because the improvement of Chinese subjects often requires students to invest large amounts of time and effort to accumulate, which is very related to students’ interest and not much related to the strength of cognitive ability; while Mathematics and English are subjects that can be improved if students follow the plan carefully. Therefore, the Self-monitoring of planning, self-control, persistence and daring are very helpful in helping students to learn according to the teacher’s requirements, and the stronger the cognitive ability, the more effective the learning will be, resulting in higher Academic Achievement.

The simple slope test reveals that self-monitoring is a consistent mechanism in moderating overall academic performance, math performance, and English performance. It is that the moderating effect is significantly greater in high self-monitoring ability than in low self-monitoring ability. Students with high levels of self-monitoring have more knowledge about learning and learning strategies, and are good at controlling their own learning process, using various strategies to solve problems, and promoting the development of strong learning skills. Students with low levels of self-monitoring, on the other hand, lack knowledge about learning and learning strategies, and are not good at using different strategies according to the changes in materials and learning tasks, and usually show poor learning ability. Good self-monitoring skills enable individuals to acquire, organize and use information more effectively and avoid losses due to low cognitive ability. On the contrary, if the cognitive ability is high but the self-monitoring level is low, the lack of self-management and self-regulation of cognitive activities makes the cognitive activities lack planning and purpose, resulting in low efficiency of problem solving and lower possibility of success. The higher the level of self-monitoring, the better it is for improving learning efficiency, bringing into play the strengths of cognitive ability, and thus achieving better academic success.

### Limitations and future directions

One of the most prominent limitations of this study is the small sample size and the single range of students surveyed. To further enhance the credibility of the study’s findings, more schools in other Chinese provinces should be selected for the study and comparison. Furthermore, this study considered only the external effects caused by self-monitoring when analyzing the effects of cognitive ability on continued academic performance. As well, the effects of students’ other psychological states on cognitive ability and Academic Achievement were not considered in the analysis. Future research could focus on this area to obtain more valuable findings.

## Conclusion

This research used structural equation modeling to analyze the moderating effect of self-monitoring of cognitive ability on academic achievement. The results of the research showed that cognitive ability can positively influence academic achievement significantly, while Self-monitoring can significantly moderate the effect of cognitive ability on academic performance, with a significant moderating effect on math subjects and English subjects among achievement subjects, and the higher the value of cognitive ability, the stronger the moderating effect.

## Data availability statement

The original contributions presented in this study are included in the article/Supplementary material, further inquiries can be directed to the corresponding author.

## Ethics statement

The studies involving human participants were reviewed and approved by the Research Ethics Committee of the School of Humanities and Social Sciences, University of Science and Technology Beijing. Written informed consent to participate in this study was provided by the participants’ legal guardian/next of kin.

## Author contributions

YS contributed to the conception and design of the study and performed the statistical analysis. YS and SQ contributed to the data collection and wrote the first draft of the manuscript. Both authors contributed to manuscript revision, read, and approved the submitted version.
